# Malic enzyme 2 promotes the progression of hepatocellular carcinoma via increasing triglyceride production

**DOI:** 10.1002/cam4.4209

**Published:** 2021-08-24

**Authors:** Shuai Zhang, Zhi‐Mei Cheng, Jia‐LI Yu, Kai Lu, Sheng‐Jie Xu, Yuan Lu, Ting Liu, Bai‐Juan Xia, Zhi Huang, Xu‐Ya Zhao, Wei He, Jun‐Xiang Li, Wei Cao, Yu Huang, Ling Wang, Zhu Zeng, Xun Zou, Rong Liu, Yu‐Sui Zhang, Xiao‐Ping Wu, Tian‐Peng Jiang, Shi Zhou

**Affiliations:** ^1^ Department of Interventional Radiology The Affiliated Cancer Hospital of Guizhou Medical University Guiyang China; ^2^ Department of Interventional Radiology The Affiliated Hospital of Guizhou Medical University Guiyang China; ^3^ Guizhou Provincial Key Laboratory of Pharmaceutics Guizhou Medical University Guiyang China; ^4^ School of Basic Medical Sciences Guizhou Medical University Guiyang China; ^5^ Department of Radiology the Affiliated Hospital of Guizhou Medical University Guiyang China; ^6^ Department of Interventional Radiology First Affiliated Hospital of Guizhou University of Traditional Chinese Medicine Guiyang China

**Keywords:** HCC, ME2, migration, triglyceride, tumorigenesis

## Abstract

The incidence and mortality of hepatocellular carcinoma (HCC) are gradually increasing during the past years. Recently, some studies have reported that malic enzyme (ME) plays an important role in cancer development, while the involvement of ME2 in HCC remains still undetermined. Here, we demonstrated that ME2 played an oncogenic role in HCC. ME2 was overexpressed in HCC tissues. TCGA database showed that the ME2 transcript level was inversely associated with the survival of HCC patients. Loss‐of‐function and gain‐of‐function assays showed that ME2 promoted HCC cell growth and migration. Furthermore, the xenografted tumorigenesis of MHCC97H cells was retarded by ME2 knockdown. ME2 silencing also suppressed the cell cycle process and induced apoptosis. Mechanistically, ME2 potentiated triglyceride synthesis, inhibition of which suppressed the proliferation and migration. We propose that ME2 promotes HCC progression by increasing triglyceride production.

## Graphical abstract

The expression of ME2 was significantly elevated in HCC tissues.ME2 promotes HCC progression by increasing triglyceride production.

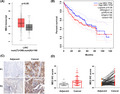

## 1 INTRODUCTION

The incidence of hepatocellular carcinoma (HCC) is increasing in Western countries because obesity is growing during the current decade.^1^ Till now, surgical approaches remain the conventional option for HCC patients regardless of great advances in diagnosis and treatment that have been obtained.^2^A mounting evidence indicates that altered cellular metabolism is a major hallmark for cancer,^3^ in which the homeostasis of metabolic fluxes in tumor cells is pivotal for proliferation, survival, and other major processes of tumorigenesis.^4^, ^5^, ^6^ It is been reported that the multiple oncogenic pathway molecules are involving in cellular metabolism. Oncogenes such as Ras, c‐Myc, and HIF1α are involving in the promotion of cancer metabolic alterations, whereas tumor suppressors such as p53 are maintaining the stability of cellular metabolism.^7^, ^8^, ^9^, ^10^, ^11^ However, few connections between cellular metabolism and the development of HCC have been established.

The chromosomal region 18q21, which contains multiple housekeeping genes, including mitochondrial malic enzyme 2 (ME2), is always homozygously depleted with SMAD4, in a large fraction of human solid tumors.^12^ The malic enzymes represent a family of oxidative decarboxylases that catalyze the conversion of malate to yield CO_2_ and pyruvate, accompanied by the production of dinucleotide cofactor NAD^+^ or NADP^+^.^13^ Malic enzymes, which comprise ME1, ME2, and ME3, are tetrameric proteins with highly conserved amino acid sequences and structures.^14^, ^15^ Comparing to ME3, ME2 is more abundant in mitochondria and has a more profound effect on the cellular metabolism of NADPH, lipid, and glutamine.^16^ Recent studies indicated that ME2 was dysregulated in several tumors and serves as a target in the regulation of tumor proliferation, differentiation, metabolism, and invasion in various cancers, such as lung cancer, pancreatic cancer, and erythroleukemia.^12^, ^17^, ^18^ Ren, J. G. et al suggested that ME2 knockdown in K562 cells leads to erythroid differentiation in chronic myelogenous leukemia.^17^ Furthermore, another study clarified that ME2 depletion would inhibit lung cancer growth via inactivation of the PI3K/AKT axis.^18^

Herein, we intended to dissect the significance of ME2 in HCC development. The expression level of ME2 was determined in human HCC tissues. Then the biological function and underlying mechanism of ME2 in HCC progression were investigated using gain and loss of function strategies. We demonstrated that ME2 functions as an important player in HCC cell proliferation, migration, and serves as a potential target for HCC diagnosis and therapy.

## 2 MATERIALS AND METHODS

### 2.1 Materials

FASN inhibitor orlistat was purchased from Roche. Human HCC cell lines Bel7402, MHCC97H, and SKHEP1 were purchased from the American Type Culture Collection (ATCC). Cell culture was kept in DMEM medium (HyClone), containing 10% FBS (Gibco), and 1% antibiotics solution (Corning). HCC microarray was purchased from SHANGHAI OUTDO BIOTECH CO., LTD. A total of 150 microarrays, including 75 HCC tissues and the adjacent normal tissues, were used for immunohistochemical staining.

### 2.2 Analysis of ME2 from TCGA

The ME2 transcript in HCC and normal tissues was analyzed from http://cancergenome.nih.gov.

### 2.3 Immunohistochemical analysis of ME2 in HCC microarrays

The paraffin‐embedded HCC microarrays were first subjected to deparaffinization, antigen retrieval, and endogenous peroxidase blockage. After blocking with 10% serum, the microarrays were incubated with ME2 (Abcam, ab139686, 1:200) antibody at 4℃ overnight and with rabbit secondary antibody for half an hour. The staining was performed using DAB.

### 2.4 ME2 over‐expression in HCC cells

The coding of the ME2 DNA sequence was cloned between BamHI and NotI site of pCDH vectors. pCDH (pCDH‐empty or pCDH‐ME2), as well as PSPAX2 and PDM2G, were transfected into the 293FT cells. Lentivirus was harvested and filtered before infection. Then the HCC cells were infected with the virus for 10 days (three days per time).

### 2.5 ME2 knockdown in HCC cells

pLL3.7 lentivirus vectors were used to knockdown ME2 in HCC cells. The lentivirus was packaged in 293FT cells when co‐transfected with pLL3.7, VSVG, REV, and pMDL vectors. HCC cells were infected by lentivirus for 10 days (three days per time). The target sequences are listed as follows: ME2#1: GCCTTACGATTTCATAGAAAC; ME2#2: GCAGGAATACGGCCTGATAGA. Knockdown of ME2 was measured by Western blot.

### 2.6 Cell proliferation assay

Cell viability was analyzed using a CCK kit. In brief, a total of 2000 HCC cells were seeded in 100 ul of DMEM medium into the 96‐well plates. At the indicated time, cells were incubated with 10 ul of CCK solution for 3 hours before measuring OD450 value. Cell viability was normalized to the OD value of day 1*100%.

### 2.7 Colony growth assessment

Ctrl and ME2 over‐expressed, shCtrl, shME2#1 and shME2#2 Bel7402, MHCC97H and SKHEP1 cells were plated at equal amount in 6‐well plates. Seven or 10 days later, each well was washed by PBS and the colonies were immobilized by methanol. Cell colony images were taken using the camera after staining the colonies with 0.1% crystal violet.

### 2.8 Xenografted tumorigenesis

10^6^ shCtrl and shME2#1 MHCC97H cells were subcutaneously transplanted into the nude mice (4–6 weeks old, female BALB/c). The tumors were initiated on day 12 after implanting. The tumors were collected and pictured at day 45. The tumors were subjected to H&E staining and immunohistochemical analysis of Ki‐67 (Abcam, ab15580, 1:500).

### 2.9 Transwell

Bel7402 or SKHEP1 cells (3 × 10^4^ cells/well for ME2 over‐expression or 6 × 10^4^ cells/well for ME2 knockdown) in FBS‐free DMEM were plated onto the upper surface of the 24‐well Transwell unit (Corning). DMEM with 10% FBS was added to the plates. The plates were incubated at 37℃ for 24 h. The units were washed with PBS three times before removing the upper cells. Subsequently, migrated cells were fixed with methyl alcohol and stained with 0.1% crystal violet. Migrated cells were pictured under a microscope.

### 2.10 Western blot

HCC cells were lysed in RIPA lysis buffer. Protein concentration was measured by BCA assay. 30–80 μg proteins were loaded on SDS‐PAGE gels and immunoblotted onto PVDF membranes. Subsequently, the membranes were incubated with 5% skim milk, which indicated primary and secondary antibodies. The chemiluminescent kit (Thermo Scientific) was used to detect the protein abundance. Antibody information was listed as follows: ME2 (Abcam), β‐actin, and secondary antibodies (Santa Cruz).

### 2.11 Quantitative real‐time PCR (qRT‐PCR)

RNA extraction was conducted using TRIzol (Invitrogen). Reverse transcription was conducted using M‐MLV (Promega). qPCR was performed on a Bio‐Rad CFX instrument with qPCR SuperMix (TransGen Biotech). The primer sequences contained E‐cadherin forward, 5′‐CGAGAGCTACACGTTCACGG‐3′, and reverse, 5′‐GGGTGTCGAGGGAAAAATAGG‐3′; N‐cadherin forward, 5′‐TGCGGTACAGTGTAACTGGG‐3′, and reverse, 5’‐GAAACCGGGCTATCTGCTCG‐3’; GAPDH forward, 5′‐TGACTTCAACAGCGACACCCA‐3′, and reverse, 5′‐CACCCTGTTGCTGTAGCCAAA‐3′. GAPDH served as the internal control.

### 2.12 Cell cycle analysis

Cell cycle distribution in shCtrl and shME2#1 Bel7402,MHCC97H and SKHEP1 cells were analyzed using Propidium iodide (PI) staining. Briefly, shCtrl and shME2#1 HCC cells were fixed with 70% ethanol and the nuclei of HCC cells were stained with PI. Subsequently, cell cycle distribution was analyzed on the flow cytometry.

### 2.13 Caspase 3/ caspase 7 activity

Caspase‐Glo reagent (Promega) was used for caspase 3/ caspase 7 activity detection. HCC cells (10^4^ cells/well) were plated into 96‐well plate and Caspase‐Glo reagent was prepared and added into each well (100 μl/well). 90 minutes later, the activity of caspase 3/caspase 7 was detected by a microplate reader.

### 2.14 Triglyceride measurement

Triglyceride content in HCC cells was measured using the kit from the APPLYGEN company. The relative triglyceride level was normalized to cell number or tissue weight.

### 2.15 Statistical analysis

The data presented as mean ±SD were analyzed with GraphPad Prism 6.0 software. Student's *t* test was performed to analyze the difference between the two groups. *p* < 0.05 was recognized as statistically significant.

## 3 RESULTS

### 3.1 ME2 is highly expressed in HCC specimens and predicts the poor survival of HCC patients

**FIGURE 1:**
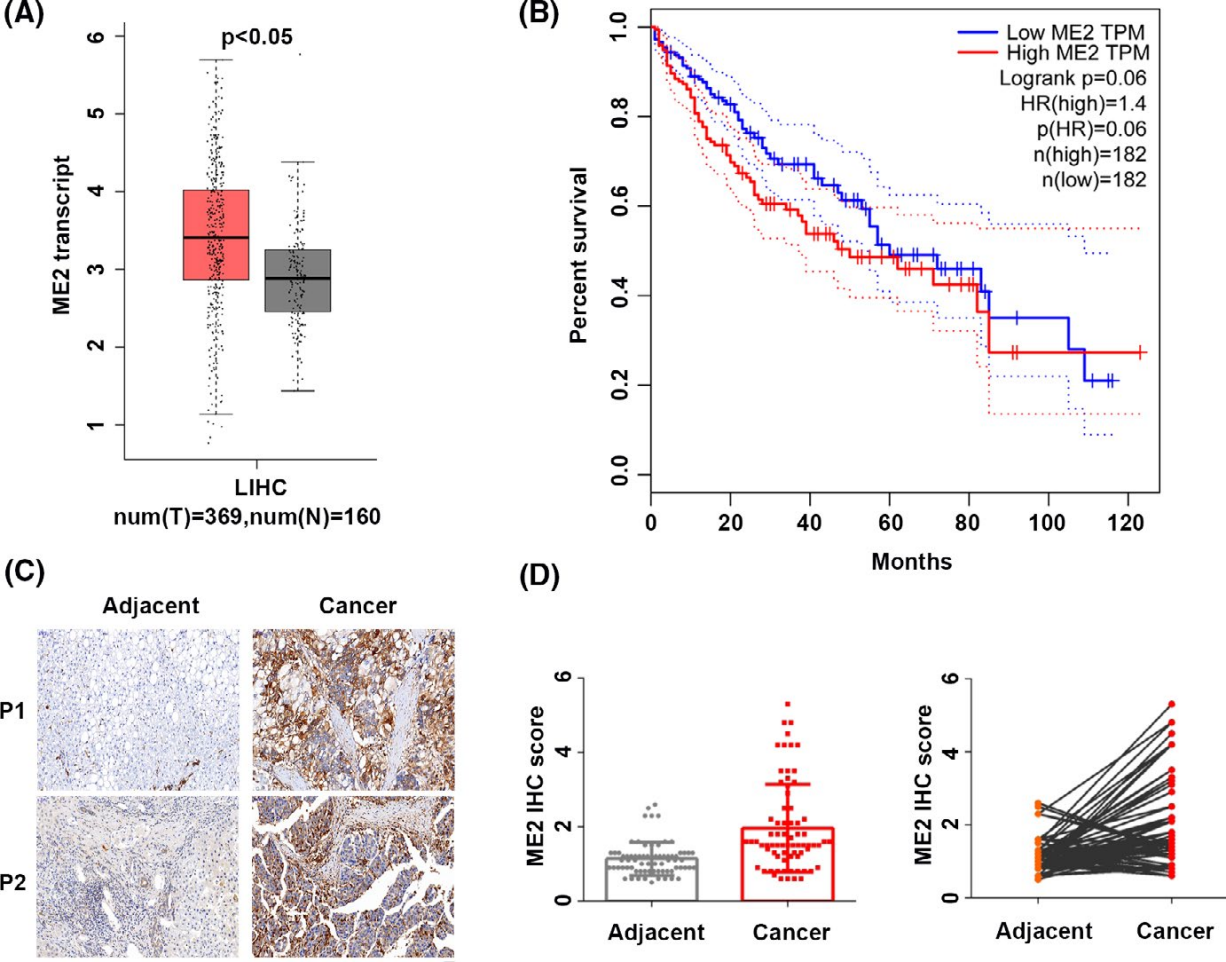
Clinical significance of ME2 in HCC. (A) ME2 transcript abundance was analyzed in HCC tissues from the TCGA. *p *< 0.05. The red represented tumor samples (*n* = 369) and the grey indicated normal samples (*n* = 160). (B) Correlation between ME2 and HCC patients’ survival was analyzed from TCGA. *p* = 0.06. (C) Immunohistochemical analysis of ME2 in HCC microarrays. *n* = 75 in each group. Scale bar, 50 µm. (D) Quantification results of IHC staining as shown in (C). *p *< 0.01

We first analyzed the clinical significance of ME1, ME2, and ME3 in HCC based on TCGA. ME1 was highly expressed in HCC samples and its overexpressed conferred poor prognosis of the patients (Figure S1A). In contrast, there was no difference in ME3 expression between normal and cancer tissues. The ME3 expression level was not correlated with patients’ survival (Figure S1B). It has been shown that ME1 promotes HCC development, while the involvement of ME2 in HCC is unknown. We then analyzed the clinical relevance of ME2 in HCC based on TCGA. Comparing with normal samples, ME2 expression was higher in HCC tissues (Figure 1A). Even though HCC patients of ME2 high expression exhibited slightly poorer survival as compared with the patients of ME2 low expression, the difference between the two groups was not significant (Figure 1B). To validate the significance of ME2 in HCC, we collected HCC samples and subjected them to IHC staining of ME2. ME2 expression was dramatically enhanced in HCC tissues comparing to their adjacent normal tissues (Figure 1C,D). Collectively, ME2 is a potential oncogene in HCC.

### 3.2 Up‐regulated ME2 promotes the growth of HCC cells

**FIGURE 2:**
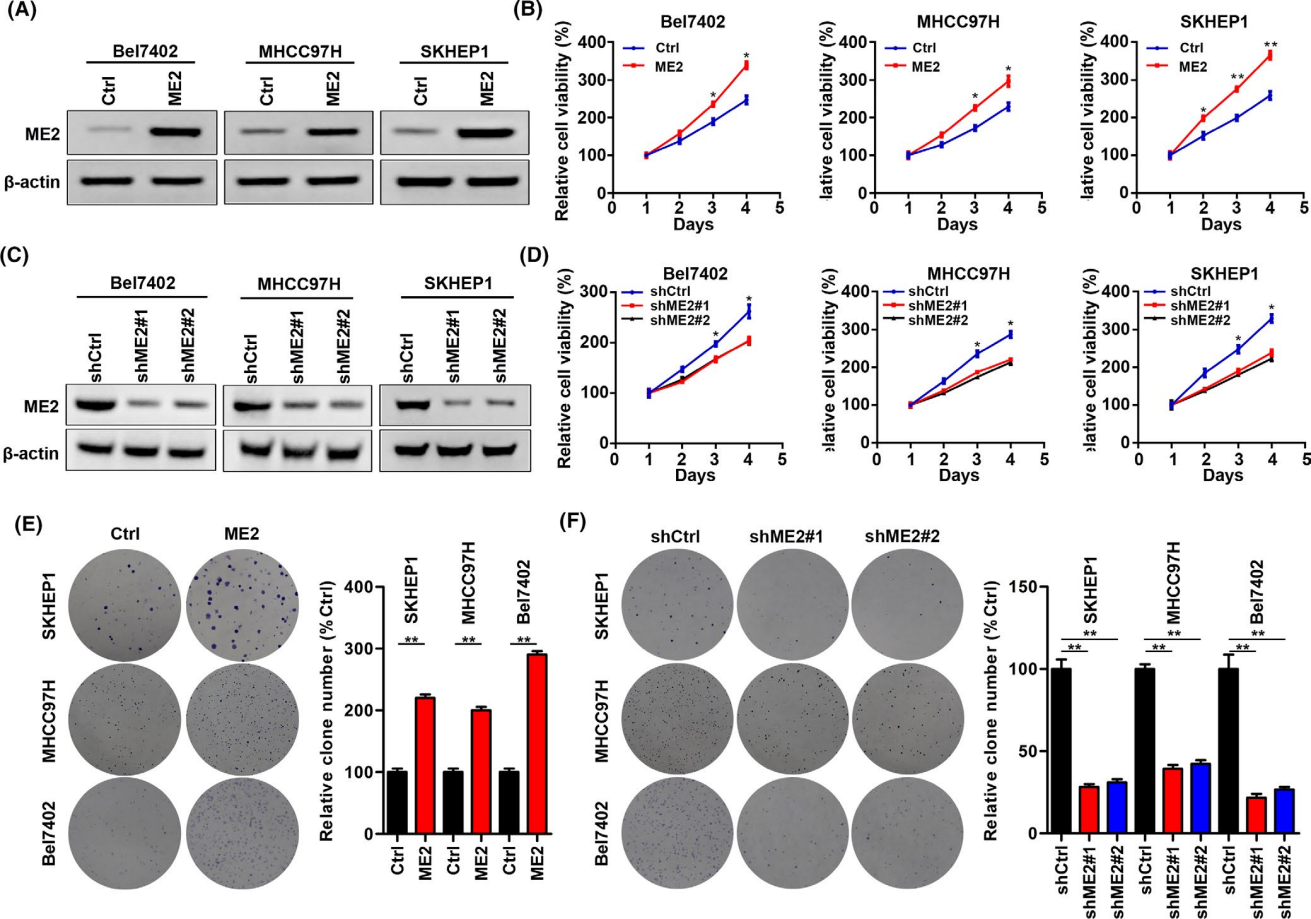
ME2 promotes HCC cell growth. (A) Immunoblotting of ME2 in Ctrl and ME2 overexpressed Bel7402, SKHEP1, and MHCC97H cells. A total of 30 µg proteins were loaded. ME2 primary antibody was diluted at 1:1000 and was incubated for 8 h at 4℃. (B) Cell growth was assessed by CCK in cells described in (A). (C) Immunoblotting of ME2 in shCtrl, shME2#1 and shME2#2Bel7402, SKHEP1 and MHCC97H cells. β‐actin serves as an internal control. A total of 60 µg proteins were loaded. ME2 primary antibody was diluted at 1:500 and was incubated for 12 h at 4℃. (D) Cell growth was assessed by CCK in cells described in (C). (E) Cell colonies were measured in cells described in A. A total of 500 SKHEP1 cells, 3000 MHCC97H cells, and 500 Bel7402 cells were seeded. (F) Cell colonies were measured in cells described in (C). A total of 500 SKHEP1 cells, 6000 MHCC97H cells, and 2000 Bel7402 cells were seeded. The experiments were performed for three independent repeats. *p < 0.05, **p < 0.01

Next, we explored the role of ME2 in HCC development using lentivirus‐mediated ME2 over‐expression and knockdown. Western blot results showed that ME2 was significantly overexpressed in HCC cells, including Bel7402, SKHEP1, and MHCC97H (Figure 2A). CCK results showed that ME2 ectopic expression accelerated the proliferation of HCC cells (Figure 2B). Moreover, we used two shRNA targets to knock down ME2 in HCC cells. Both shRNAs efficiently suppressed the expression of ME2 (Figure 2C). ME2 silencing inhibited the proliferation of HCC cells (Figure 2D). Consistently, the colony formation of HCC cells was enhanced and reduced by ME2 over‐expression and knockdown, respectively (Figure 2E,F). Taken together, ME2 is essential to the proliferation and growth of HCC cells.

### 3.3 ME2 silencing suppresses the xenografted tumorigenesis of MHCC97H cells

**FIGURE 3:**
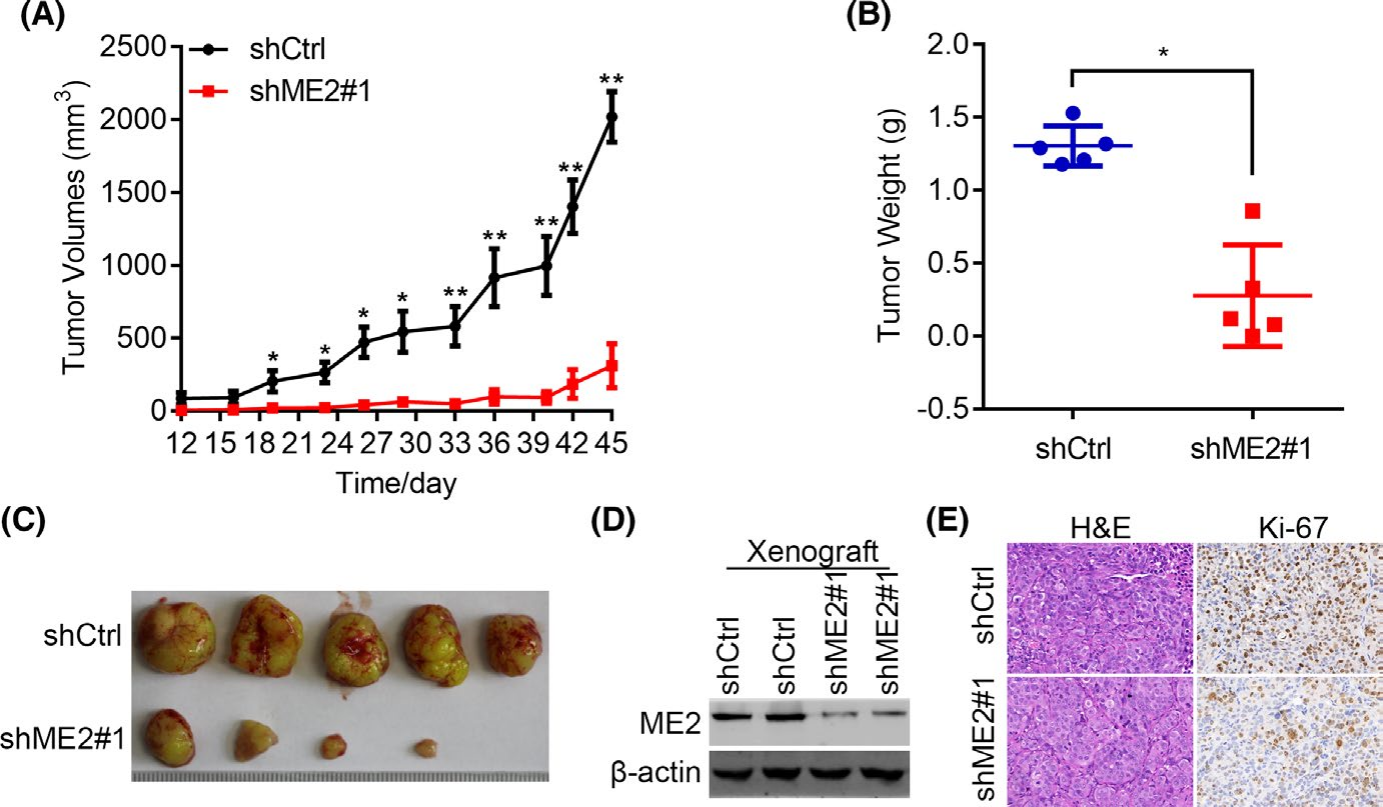
ME2 knockdown suppresses the xenografted tumor development of MHCC97H cells. (A) The growth curve of tumors derived from the nude mice implanted with shCtrl and shME2#1 MHCC97H cells. (B) Tumor weight was measured. (C) Photographs of the tumors as described in (A). (D) Immunoblotting of ME2 in shCtrl and shME2#1 xenografted tumors. A total of 40 µg proteins were loaded. (E) Ki‐67 IHC staining of tumors as described in (A). Scale bar, 50 µm. *p < 0.05, **p < 0.01

To study the *in vivo* role of ME2 in HCC development, shCtrl, and shME2#1 MHCC97H cells were subcutaneously transplanted into the BALB/c nude mice. ME2 knockdown significantly suppressed the tumor initiation and progression of MHCC97H cells (Figure 3A–C). Immunoblotting results demonstrated that ME2 was downregulated in tumors derived from shME2#1 MHCC97H cells (Figure 3D). Then the tumors derived from shCtrl and shME2 MHCC97H cells were subjected to IHC staining of Ki‐67, a well‐known proliferation marker. The results showed that ME2 knockdown largely inhibited the proliferation of HCC cells *in vivo* (Figure 3E). Our findings suggest that ME2 is critical for the tumor growth of HCC cells.

### 3.4 ME2 potentiates the EMT transition and the migration of HCC cells

**FIGURE 4:**
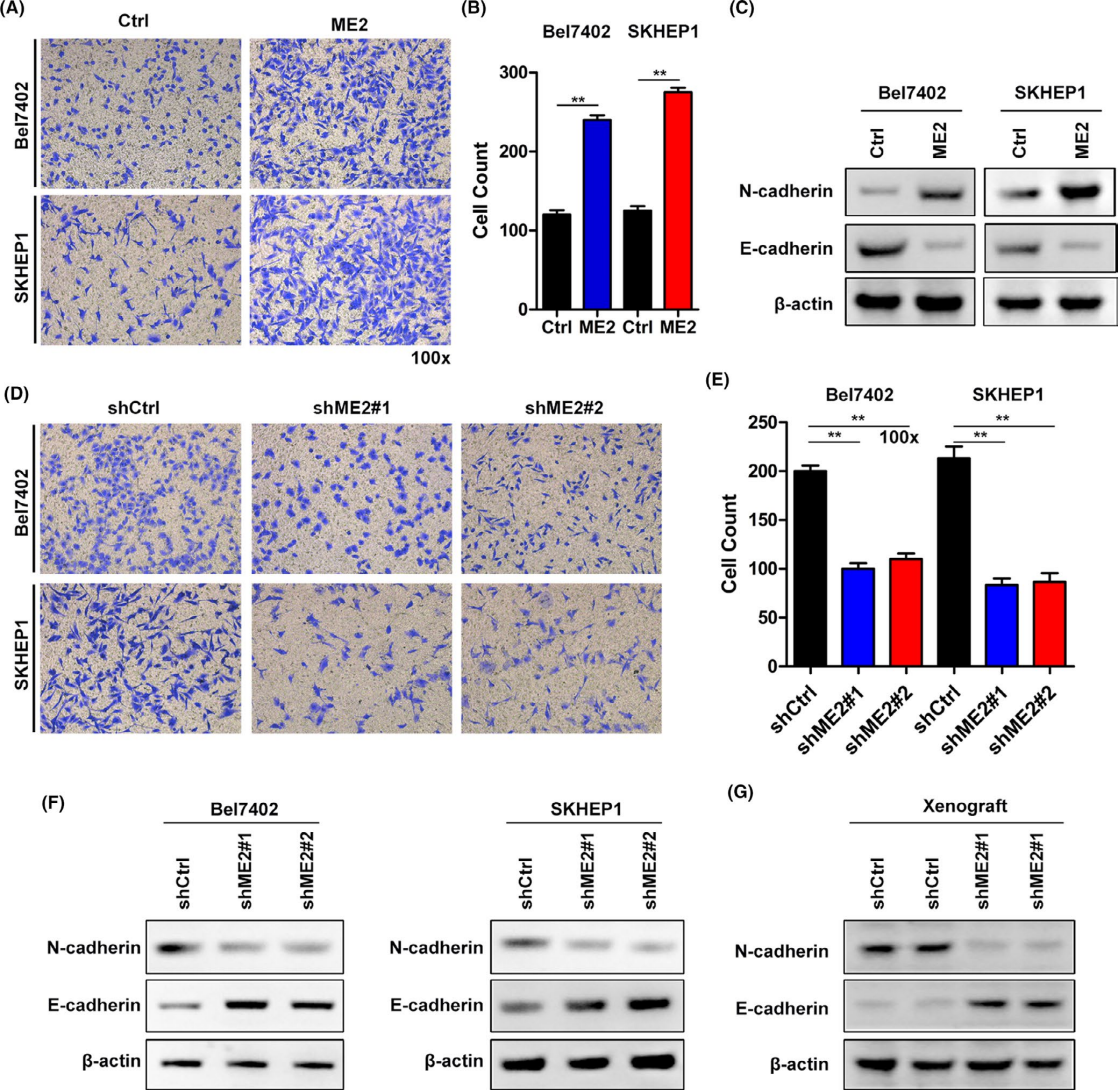
ME2 promotes the migration of HCC cells. (A,B) Migration was assessed by Transwell in Ctrl and ME2 overexpressed Bel7402 and SKHEP1 cells. A total of 3 × 10^4^ cells/well were seeded. (C) Immunoblotting of N‐cadherin and E‐cadherin in Ctrl and ME2 overexpressed Bel7402 and SKHEP1 cells. A total of 50 µg proteins were loaded. N‐cadherin primary antibody was diluted at 1:1000 and was incubated for 8 h at 4℃. E‐cadherin primary antibody was diluted at 1:500 and was incubated for 12 h at 4℃. (D,E) Cell migration was measured by Transwell in shCtrl, shME2#1 and shME2#2Bel7402, and SKHEP1 cells. A total of 6 × 10^4^ cells/well were seeded. (F,G) Immunoblotting of N‐cadherin and E‐cadherin in shCtrl, shME2#1, and shME2#2 Bel7402 or SKHEP1 cells (F) and in shCtrl and shME2#1 xenografted tumors (G). For (F), a total of 50 µg proteins were loaded. N‐cadherin primary antibody was diluted at 1:500 and was incubated for 12 h at 4℃. E‐cadherin primary antibody was diluted at 1:2000 and was incubated for 8 h at 4℃. For (G), a total of 50 µg proteins were loaded. N‐cadherin primary antibody was diluted at 1:500 and was incubated for 12 h at 4℃. E‐cadherin primary antibody was diluted at 1:2000 and was incubated for 8 h at 4℃. The experiments were performed for three independent repeats. **p < 0.01

HCC is a malignancy with high frequency of metastasis. We then investigated whether ME2 regulated the migration of HCC cells. To address this question, we performed Transwell experiments in ME2 overexpressed or silenced Bel7402 and SKHEP1 cells. This assay was not performed in MHCC97H cells because they could not migrate through the well. We observed that ME2 ectopic expression enhanced the migration capacity of both Bel7402 and SKHEP1 cells (Figure 4A,B). Opposite results were observed in ME2 knockdown Bel7402 and SKHEP1 cells (Figure 4D,E). We also found that ME2 promoted the expression of N‐cadherin and suppressed the expression of E‐cadherin in Bel7402 and SKHEP1 cells (Figure 4C,F). In addition, the shME2 xenografted tumors had reduced N‐cadherin and increased E‐cadherin expression as compared with shCtrl tumors (Figure 4G). Collectively, ME2 regulates EMT transition and migration of HCC cells.

### 3.5 ME2 knockdown promotes cell cycle arrest and apoptosis

**FIGURE 5:**
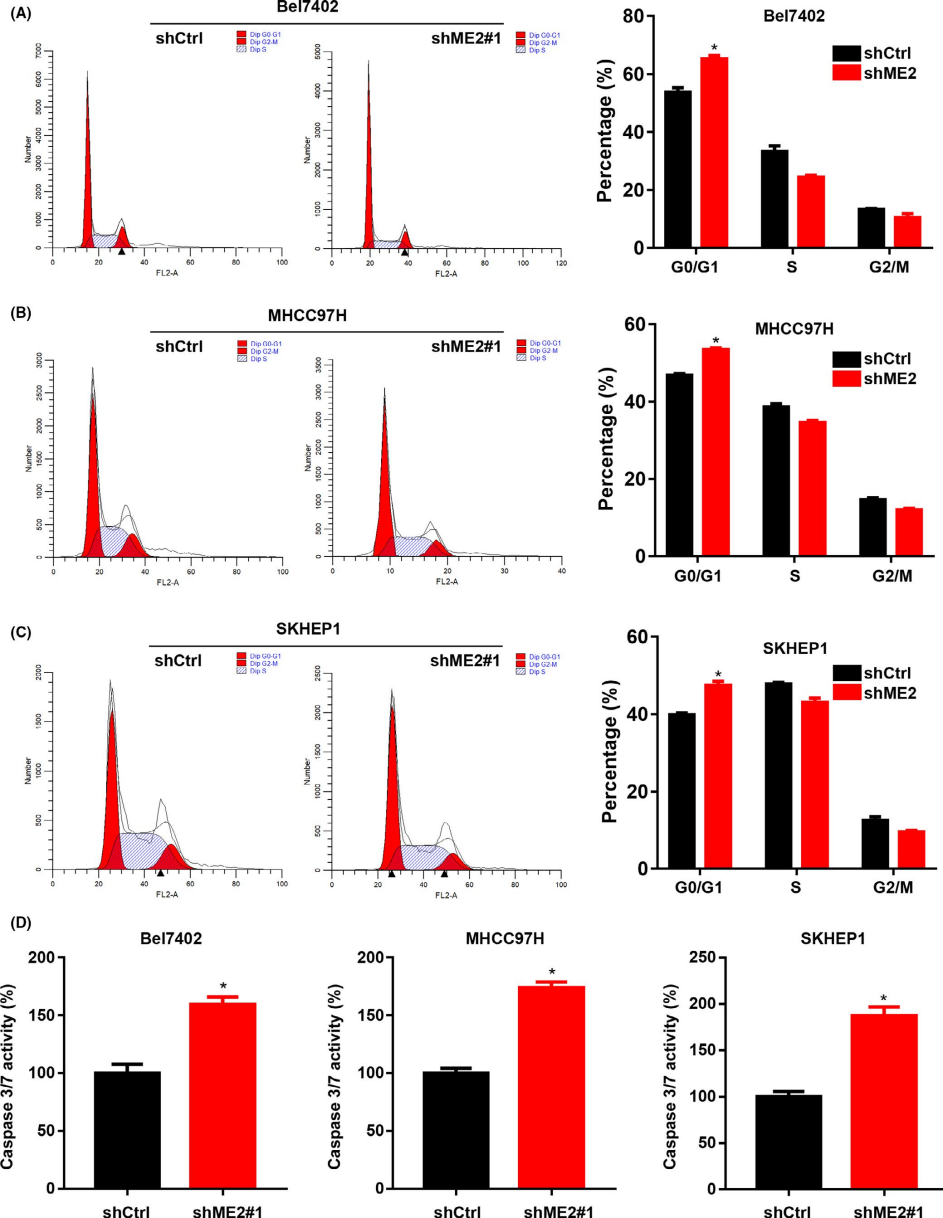
ME2 knockdown induces apoptosis and suppresses cell cycle progression. (A–C) Cell cycle distribution was analyzed in shCtrl, shME2#1, and shME2#2 Bel7402 (A), SKHEP1 (B), and MHCC97H (C) cells. Left, representative images; Right, quantitative results. (D) Caspase 3/ caspase 7 activity was analyzed in Bel7402, SKHEP1, and MHCC97H cells. *p < 0.05

Suppressed apoptosis and uncontrolled cell cycle progression are the common characteristics of cancer cells. We then analyzed the cell cycle distribution and apoptosis in ME2 silenced HCC cells. Comparing with shCtrl HCC cells, shME2 cells exhibited increased cell number at the G0 phase (Figure 5A–C), which represents the non‐proliferative phase. Caspase 3/ caspase 7 activity was increased in ME2 knockdown Bel7402, SKHEP1, and MHCC97H cells (Figure 5D. These results indicate that ME2 knockdown leads to suppressed cell cycle progression and enhanced apoptosis.

### 3.6 ME2 positively regulates triglyceride production in HCC cells

**FIGURE 6:**
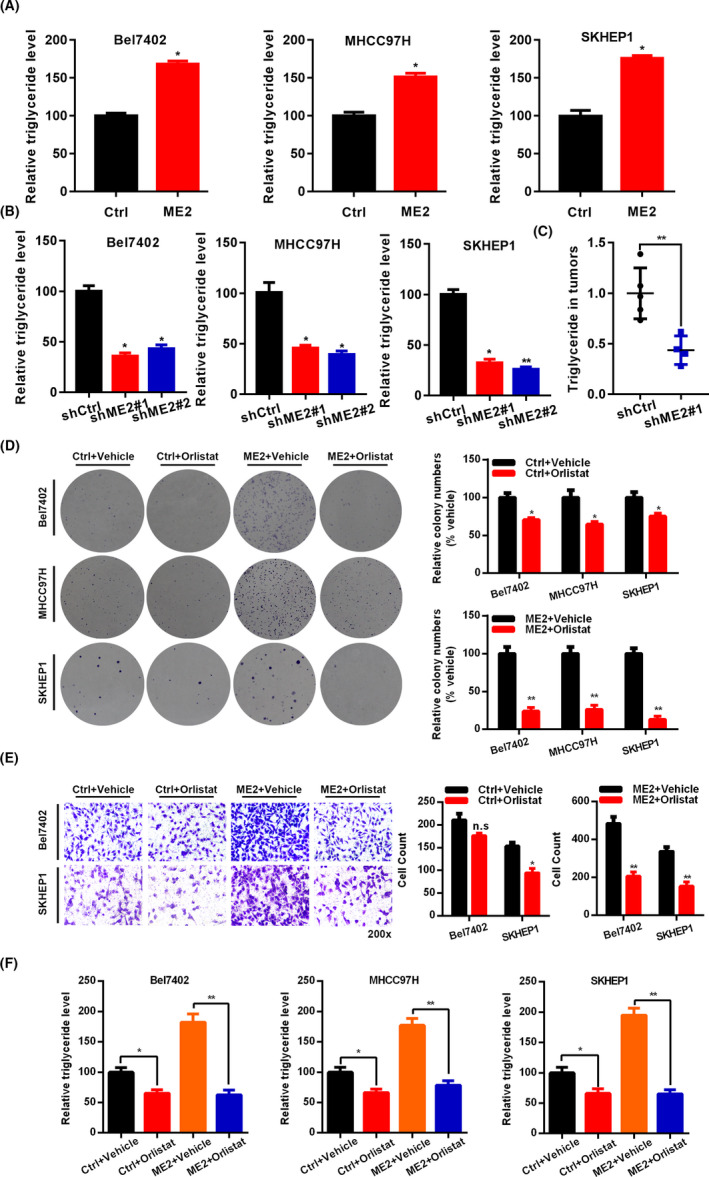
ME2 increases triglyceride synthesis to potentiate cell growth and migration. (A)Triglyceride content was measured in Ctrl and ME2 overexpressed Bel7402, SKHEP1, and MHCC97H cells. (B) Triglyceride content was measured in shCtrl, shME2#1, and shME2#2 Bel7402, SKHEP1, and MHCC97H cells. (C) Triglyceride content was measured in shCtrl (*n* = 5) and shME2#1 (*n* = 4) xenografted tumor tissues. (D) Colony growth was measured in Ctrl and ME2 overexpressed Bel7402, SKHEP1, and MHCC97H cells which were treated with or without FASN inhibitor. (E) Ctrl and ME2 overexpressed Bel7402, SKHEP1, and MHCC97H cells were treated with or without FASN inhibitor orlistat and then subjected to Transwell assay. n.s, no significance, (F) Triglyceride content was measured Ctrl and ME2 overexpressed Bel7402, SKHEP1, and MHCC97H cells that were treated with or without FASN inhibitor orlistat. *p < 0.05, **p < 0.01

Malic enzyme plays a pivotal role in regulating gluconeogenesis, glycolysis, and fatty acid synthesis. Because fatty liver is the main risk factor for HCC, we next determined whether ME2 regulated lipid metabolism. Triglyceride is a key resource of liver lipids. We found that ME2 over‐expression led to increased triglyceride in Bel7402, SKHEP1, and MHCC97H cells (Figure 6A). In contrast, ME2 downregulation suppressed the triglyceride production in these cells (Figure 6B). In addition, ME2 knockdown also decreased triglyceride content in tumor tissues (Figure 6C). These results suggest that ME2 enhances triglyceride synthesis in HCC cells.

### 3.7 Inhibition of triglyceride synthesis suppresses the biology function of HCC cells

FASN is an essential regulator of fatty acid synthesis. To explore whether ME2 elevation of triglyceride synthesis is critical for HCC development, we treated the Ctrl and ME2 overexpressed HCC cells with FASN inhibitor orlistat and subjected them to colony formation and Transwell analysis. Orlistat significantly suppressed the growth of ME2 overexpressed Bel7402, SKHEP1, and MHCC97H cells, but had a minimal inhibitory effect on the colony growth of Ctrl cells (Figure 6D). Cell cycle progression was more significantly inhibited by orlistat in ME2 overexpressed cells as compared with Ctrl cells (Figure S2A). The migration of Ctrl Bel7402 and SKHEP1 cells, as well as EMT in Bel7402 cells, was not significantly inhibited by orlistat. However, orlistat suppressed the migration capacity of Bel7402 and SKHEP1 cells, as well as EMT of Bel7402 cells, with ectopic expression of ME2 (Figure 6E and Figure S2B). In addition, orlistat led to a larger reduction of triglyceride abundance in ME2 overexpressed cells as compared in Ctrl cells (Figure 6F). Interestingly, ME2 expression was positively correlated with the triglyceride content in human HCC/normal samples (Figure S2C). Therefore, ME2 promotes HCC development at least partly by enhancing the triglyceride production.

## 4 DISCUSSION

HCC is one of the most lethal tumors, characterized by high heterogeneity and poor prognosis, accounting for approximately 700,000 deaths per year.^19^ The main treatment is surgical resection and the 5‐year overall survival rate is nearly 70%, while the recurrence is pretty high.^20^ Thus, understanding the pathogenesis of HCC and identifying novel biomarkers are very important for the early diagnosis of HCC. Recent experimental findings have shed light on the strong correlation between reprogramming of energy metabolism and tumor development, and intend to obtain new ways for effectively controlling tumor cells by regulating their energy metabolism.^21^ Among multiple molecules and signals involving in cellular metabolism, ME2 was suggested to play crucial roles in modulating cell growth, proliferation, metabolism, and invasion of many types of cancer.^22^, ^23^, ^24^

Malic enzyme (ME) family comprises three members, including ME1, ME2, and ME3. These three enzymes play an important role in regulating the cellular metabolism of pyruvate to the TCA cycle. Recent studies have shown that ME1 contributes to the progression of several cancers, such as oral squamous cell carcinoma,^25^ breast cancer,^26^ colorectal cancer,^27^ gastric cancer,^28^, ^29^ lung cancer,^30^ and nasopharyngeal carcinoma.^31^ ME1 is also correlated with lipogenesis and HCC.^32^, ^33^, ^34^ Previous studies have investigated that ME2 is significantly dysregulated and correlates with tumor progression. Prasenjit Dey et al have revealed that the expression of ME1 is critical for the survival of ME2 depleted pancreatic cancer cells.^12^ Another study suggests that the downregulation of ME2 reciprocally activates p53 in a feed‐forward manner to induce senescence.^16^ Nevertheless, it remains obscure on the role of ME2 in HCC. The evidence in our study reveal that ME2 serves as an oncogene in HCC progression.

Hence, to explore the role of ME2, we first analyzed the clinical relevance of ME2 in HCC through the expression data from the public TCGA database. We found that ME2 was upregulated in HCC tissues. Overexpression of ME2 was closely associated with poor prognosis in the patients. Furthermore, knockdown of ME2 resulted in suppressed proliferation and increased apoptosis, as well as inhibited the EMT process and cell migration in vitro, while opposite results were obtained upon ME2 overexpression in HCC cells. Also a striking phenotype that the abolished growth of HCC cells in nude mice was observed upon ME2 knockdown, which indicated that ME2 functioned as an oncogene in HCC development.

ME2 is reported to produce a local pyruvate pool and to supply pyruvate to the TCA cycle.^35^ Since silencing ME2 may alter TCA cycle metabolism and cause malate accumulation and pyruvate decrease, which will change growth in lung cancer cells,^18^ we next sought to explore the underlying mechanism involved in the action of ME2 on cell proliferation and migration in HCC. First, we examined whether the alteration of ME2 expression would affect triglyceride metabolism, which coordinates energy production and biosynthesis.^36^ The results suggested that ME2 knockdown decreased the triglyceride level in HCC cells and vice versa. Triglyceride comprises one molecule of glycerol 3‐phosphate and three molecules of fatty acid. Fatty acid synthase (FASN) is an essential enzyme that promotes the synthesis of fatty acid. This implies FASN inhibitor could efficiently suppress the synthesis of triglyceride. Furthermore, pharmaceutical ME2 specific inhibitors were not available from pharmaceuticals companies. To validate the mechanism, we used fatty acid synthase (FASN) inhibitor orlistat instead of ME2 inhibitor to identify the influence of ME2‐mediated triglyceride metabolism on cell growth and migration. We found that the inhibition of lipid metabolism suppressed the capacity of colony formation and migration of ME2 overexpressed HCC cells. However, the colony growth and migration of Ctrl cells were not significantly retarded by orlistat. Importantly, orlistat resulted in a larger reduction of triglyceride production in ME2 overexpressed cells comparing with that in Ctrl cells. Cell cycle and EMT markers expression were also more significantly suppressed by orlistat in ME2 overexpressed cells than in Ctrl cells. Furthermore, ME2 expression was positively correlated with triglyceride abundance in HCC/normal samples. Thus, we speculated that ME2 might regulate HCC cell growth and migration partly through affecting triglyceride level, though the detailed information that ME2 might involvement in the pathway of lipid metabolism need further investigation.

Collectively, this study identified the pivotal roles of ME2 in HCC cell proliferation, apoptosis, migration, EMT process, and cell cycle, which might act as an attractive metabolic target for HCC therapy.

## 5 ETHICS STATEMENT

Animal experiments were approved by the Ethics Committee and were performed according to the animal guideline of The Affiliated Cancer Hospital of Guizhou Medical University.

## CONFLICT OF INTEREST

We declare that the authors have no conflict of interest.

## Supporting information

Fig S1‐S2

## Data Availability

All data generated in this study are available in this article.
