# Supplementary material for: Malic enzyme 2 promotes the progression of hepatocellular carcinoma via increasing triglyceride production
Source: Cancer Med. 2021 Aug 24;10(19):6795–806. doi: 10.1002/cam4.4209 (PMC8495273; doi:10.1002/cam4.4209)
Supplement: Supplementary file 1 — Fig S1‐S2 [file CAM4-10-6795-s001.docx]

**Supplementary Figure 1 Clinical significance of ME1 and ME3 in HCC.**


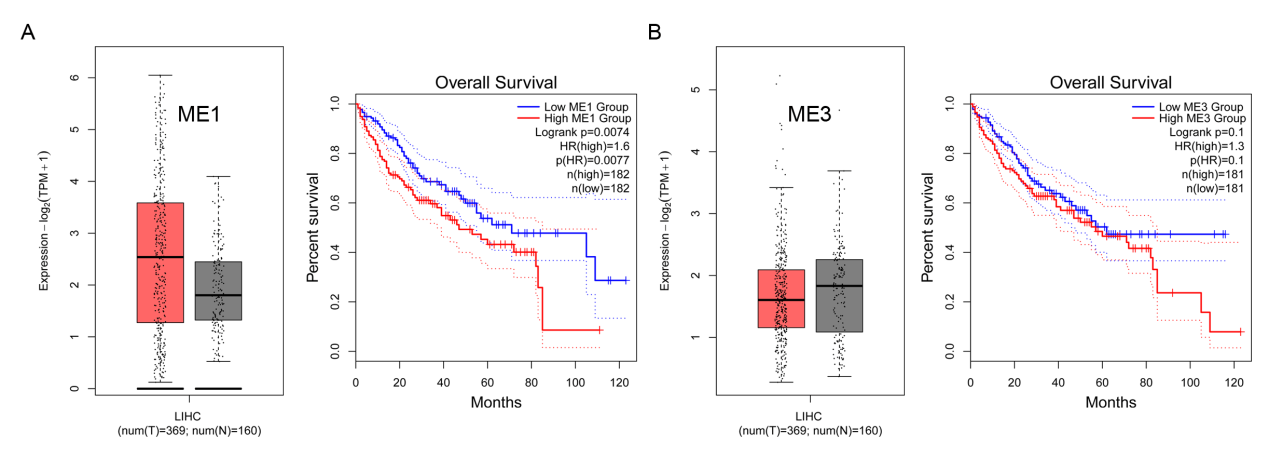


(A) The ME1 mRNA expression was analyzed in HCC tissues from the TCGA database. p<0.05. (B) The overall survival analysis of HCC patients who are divided into ME1 high expression and low expression group. p=0.0074.(C) The ME1 mRNA expression was analyzed in HCC tissues from the TCGA database. p>0.05. (D) The overall survival analysis of HCC patients who are divided into ME1 high expression and low expression group. p=0.1.

**Supplementary Figure 2 Orlistat inhibits EMT and cell cycle progression in ME2 overexpressed HCC cells.**

**
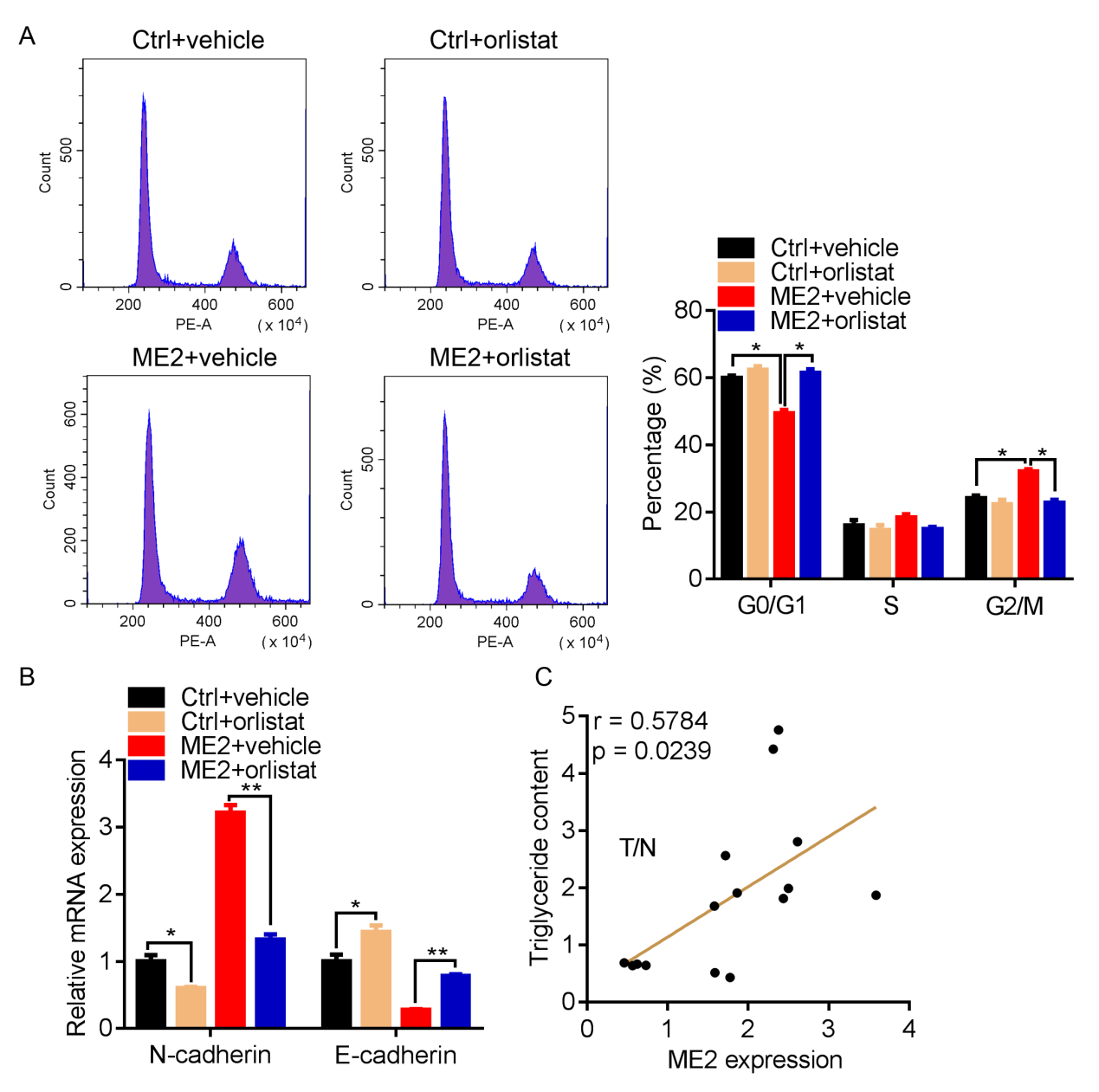
**

(A) Cell cycle distribution was measured Ctrl and ME2 over-expressed Bel7402, SKHEP1 and MHCC97H cells that were treated with or without FASN inhibitor orlistat. *p<0.05. (B) qRT-PCR analysis of N-cadherin and E-cadherin in Ctrl and ME2 over-expressed Bel7402, SKHEP1 and MHCC97H cells that were treated with or without FASN inhibitor orlistat. *p<0.05. **p<0.01. (C) Sperman correlation was analyzed between ME2 expression and triglyceride content in human HCC/normal samples. r=0.5748. p=0.0239.
